# Membrane vesicle production by *Chlamydia trachomatis* as an adaptive response

**DOI:** 10.3389/fcimb.2014.00073

**Published:** 2014-06-10

**Authors:** Kyla M. Frohlich, Ziyu Hua, Alison J. Quayle, Jin Wang, Maria E. Lewis, Chau-wen Chou, Miao Luo, Lyndsey R. Buckner, Li Shen

**Affiliations:** ^1^Department of Microbiology, Immunology, and Parasitology, Louisiana State University Health Sciences CenterNew Orleans, LA, USA; ^2^Department of Neonatology, Ministry of Education Key Laboratory of Child Development and Disorder, The Children's Hospital, Chongqing Medical UniversityChongqing, China; ^3^Department of Chemistry, University of GeorgiaAthens, GA, USA

**Keywords:** *Chlamydia trachomatis*, membrane vesicles, adaptive response, persistent growth state, stress

## Abstract

Bacteria have evolved specific adaptive responses to cope with changing environments. These adaptations include stress response phenotypes with dynamic modifications of the bacterial cell envelope and generation of membrane vesicles (MVs). The obligate intracellular bacterium, *Chlamydia trachomatis*, typically has a biphasic lifestyle, but can enter into an altered growth state typified by morphologically aberrant chlamydial forms, termed persistent growth forms, when induced by stress *in vitro.* How *C. trachomatis* can adapt to a persistent growth state in host epithelial cells *in vivo* is not well understood, but is an important question, since it extends the host-bacterial relationship *in vitro* and has thus been indicated as a survival mechanism in chronic chlamydial infections. Here, we review recent findings on the mechanistic aspects of bacterial adaptation to stress with a focus on how *C. trachomatis* remodels its envelope, produces MVs, and the potential important consequences of MV production with respect to host-pathogen interactions. Emerging data suggest that the generation of MVs may be an important mechanism for *C. trachomatis* intracellular survival of stress, and thus may aid in the establishment of a chronic infection in human genital epithelial cells.

## Introduction

*Chlamydia trachomatis* serovars D-K account for the most prevalent bacterial sexually transmitted infections (STIs) worldwide. Despite aggressive control efforts, *C. trachomatis* infections have continued to constitute a serious public health risk (World Health Organization, 2011; Rekart et al., 2013). Infection may result in cervicitis, and in some women, *C. trachomatis* may ascend into the endometrium and Fallopian tubes, where it can establish a chronic infection leading to diseases such as pelvic inflammatory disease (PID), ectopic pregnancy, and infertility. *C. trachomatis* infections of women also pose a risk to infants, as infants born from mothers with *C. trachomatis* infections can develop conjunctivitis and/or pneumonia. Finally, epidemiological evidence indicates that *C. trachomatis* infection of the reproductive tract also may increase the risk of HIV transmission, making the study and understanding of the pathogenicity of this bacterium imperative.

In adapting to an intracellular niche, *C. trachomatis* has evolved a notably reduced genome of ~1 million base pairs that supports a unique developmental cycle (Stephens et al., 1998). This cycle typically involves two forms: infectious elementary bodies (EBs) and dividing, metabolically active reticulate bodies (RBs) (Moulder, 1991). Under stressful conditions *in vitro*, however, *C. trachomatis* can enter into an alternative viable but non-dividing growth form, termed a persistent growth form (Beatty et al., 1994b). This persistent growth state extends the host-bacterial relationship *in vitro* and has thus been proposed to be linked to chronic infection and adverse outcomes, including elicitation of tissue-damaging host responses, *in vivo* (Hogan et al., 2004; Darville and Hiltke, 2010). Persistent forms are also less responsive to antimicrobial therapy *in vitro* (Wyrick and Knight, 2004; Reveneau et al., 2005) and *in vivo* (Byrne, 2001; Phillips-Campbell et al., 2014). Several excellent reviews provide extensively insightful descriptions of the chlamydial persistent growth state and the potential to establish a chronic relationship with the host (Beatty et al., 1994b; Hogan et al., 2004; McClarty et al., 2007; Wyrick, 2010; Schoborg, 2011; Lo et al., 2012).

*C. trachomatis* growth exclusively takes place within an inclusion, a membrane-bound vacuole. Despite the presence of this barrier, *C. trachomatis* actively communicates with the host cells. One method of interaction is the coordination of trafficking specific subsets of host vesicles to and from the inclusion, enabling delivery of the inclusion components and nutrients required for infection (Fields and Hackstadt, 2002). *C. trachomatis* also secretes numerous effectors with host cell-modulating activities across the complex membranes of the bacterium and the inclusion or host cytoplasm to host cell compartments. Although diverse secretory pathways, including the type II (Sec), type III, and type V (autotransporter) secretion systems, have been shown to play a role in the translocation of protein effectors (Crane et al., 2006; Valdivia, 2008; Chen et al., 2010a; Mueller et al., 2014), a mechanism for robust delivery of complex bacterial components to host cells is likely to be mediated by membrane vesicles (MVs) that emerge from the envelope of growing bacteria (Giles et al., 2006; Giles and Wyrick, 2008; Wang et al., 2011a; Frohlich et al., 2012). As part of bacterial growth and/or envelope stress responses, the formation of MVs is a universal feature found in all Gram-negative bacteria (Beveridge, 1999; Kulp and Kuehn, 2010), *Mycobacterium* spp. (Prados-Rosales et al., 2011), and Gram-positive bacteria such as *Bacillus* spp. (Dubey and Ben-Yehuda, 2011). Herein, we review recent findings on the mechanistic aspects of bacterial adaptation to stress with a focus on how *C. trachomatis* remodels its envelope, produces MVs, and the potential important consequences of MV production with respect to host-pathogen interactions.

## Molecular architecture of the cell envelope during the chlamydial developmental cycle

**Figure 1:**
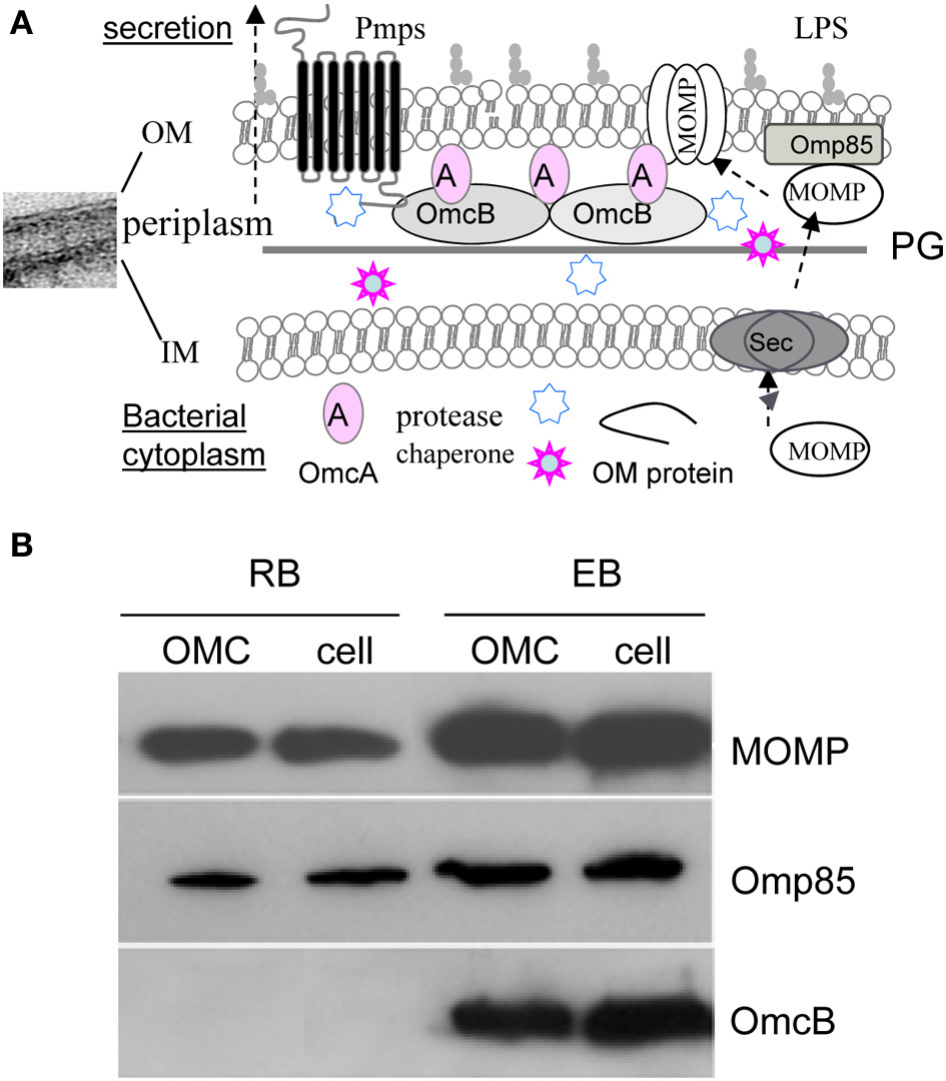
**(A)** A schematic diagram of the envelope structure of *C. trachomatis*. Showing are the representative OM associated components [Lipopolysaccharide (LPS), peptidoglycan (PG), MOMP, Pmps, OmcA, OmcB, and Omp85], and putative chaperone and protease in the periplasm in EBs. The potential process of MOMP or other OM protein transport across the inner membrane via the Sec pathway, and insertion and assembly into the OM is indicated. **(B)** Immunoblot of *C. trachomatis* serovar F Omp85. Similar to MOMP, Omp85 is present in both EBs and RBs as a component of the OM complex (OMC), however, OmcB exists only in EBs. The OMCs were prepared from the insoluble fraction of isolated EBs or RBs in phosphate buffered saline containing 2% Sarkysol as described previously (Caldwell et al., 1981). The lysates of EBs and RBs, and their OMCs were dissolved in Laemmli sample buffer (1:1, v:v) supplemented with 5% 2-mercaptoethanol, 10 mM dithiothreitol and heated for 10 min at 100°C, isolated on 10% SDS-PAGE, followed by immunoblotting using polyclonal antibodies to Omp85 or OmcB, and a monoclonal antibody to *C. trachomatis* serovar F MOMP.

One of the critical developmental events common to *Chlamydia* spp. is the ability of the organisms to adapt their envelopes for the purpose of interacting with the host cell. Like other Gram-negative bacteria, the *C. trachomatis* envelope consists of an outer membrane (OM), an inner membrane (IM), and a periplasm (Figure 1A). Recent elegant studies by several groups have revealed that chlamydiae possess functional peptidoglycan necessary for bacterial division, despite the lack of the cytoskeletal protein FtsZ (McCoy and Maurelli, 2006; Ouellette et al., 2012; Pilhofer et al., 2013; Liechti et al., 2014). Nevertheless, a unique feature of the chlamydial envelope, distinguishing them from other Gram-negative bacteria, is the presence of a disulfide bond cross-linked major outer membrane protein (MOMP) with the periplasmic localized OmcB and the lipoprotein OmcA only in EBs (Hackstadt et al., 1985; Hatch, 1996). Both OmcB and OmcA contain abundant cysteines. This cross-linkage is believed to contribute to cell wall rigidity and osmotic stability of the EBs.

Proteins of the OM are important for various purposes including envelope architecture, virulence, transport, cell division, induction of inflammatory cytokine production, and immune evasion (Hatch, 1996; Stephens and Lammel, 2001; Abdelsamed et al., 2013). All of these tasks are presumably related to the generation and function of MVs that will be discussed in the later sections of this review. MOMP is the most abundant surface-exposed protein of both RBs and EBs (Caldwell et al., 1981; Hatch, 1996) and it functions as an adherin and a porin with its β-barrel structure (Wang et al., 2006; Sun et al., 2007). MOMP also has alternative conformations that may adapt to specific chlamydial growth stages (Feher et al., 2013) and may impart different levels of immunogenicity. Also located in the OM is a family of polymorphic membrane proteins (Pmps) or autotransporters unique to *Chlamydia* spp. (Stephens et al., 1998). *C. trachomatis* encodes nine Pmps (PmpA-I) that are either temporally or constitutively expressed (Tanzer and Hatch, 2001; Tan et al., 2010). Such temporal expression may promote antigenic variation, tissue tropism, and differential disease severity (Gomes et al., 2006; Tan et al., 2009; Taylor et al., 2011; Abdelsamed et al., 2013). Caldwell's group has reported that PmpD is a species-common, pan-neutralizing target (Crane et al., 2006). Other OM proteins studied include plasmid encoded Pgp3 (Chen et al., 2010b), PorB (Kubo and Stephens, 2000), HSP70 (DnaK) (Raulston, 1995), and Omp85 (YaeT) (Stephens and Lammel, 2001) that exists in both EBs and RBs (Figure 1B) (Liu et al., 2010). In bacteria, Omp85 facilitates the insertion of assembly intermediates from the periplasm to the OM (Ricci and Silhavy, 2012).

Many chlamydial envelope components are ligands recognized by host pattern-recognition receptors that induce inflammatory cytokine production and generate adaptive immune responses (Wang et al., 2010; Taylor et al., 2011; Abdelsamed et al., 2013). Some envelope components may also contribute to immune evasion. To this end, several chlamydial envelope components have been considered vaccine candidates (Crane et al., 2006; Schautteet et al., 2011; Hafner et al., 2014). *Chlamydia* spp. may quantitatively or qualitatively change their envelope structures as a result of adaptation to developmental signals or environmental cues (Beatty et al., 1994b; Hatch, 1996; Carrasco et al., 2011). The plasticity of the envelope has important implications for the design of control strategies against bacterial infection because of its importance in determining susceptibility to host defenses and antibiotics (Tan et al., 2009; Hurdle et al., 2011).

## Stress induces dramatic changes in chlamydia spp. *in vitro*

Matsumoto and Manire described the first ultrastructure of an aberrant RB form induced by penicillin in *C. psittaci*-infected L929 cells as viewed by transmission electron microscopy (TEM) (Matsumoto and Manire, 1970). They found that, in the presence of penicillin, RBs no longer divided, the RB to EB transition was disrupted, and aberrant multinucleated RBs accumulated. Concurrently, abundant vesicles were pinched off the aberrant RBs. Removal of penicillin allowed for the chlamydiae to reenter a normal growth state. Many elegant studies since have revealed that the viable but nondividing altered persistent form occurs when *Chlamydia* spp. are exposed to a variety of stress conditions. These include IFNγ (Beatty et al., 1994a), sub-inhibitory concentrations of antibiotics (Engel, 1992; Wyrick and Knight, 2004), nutrient deprivation (Beatty et al., 1994a; Raulston, 1997; Harper et al., 2000), co-infection with either herpes simplex virus (Vanover et al., 2008; Prusty et al., 2012) or *Toxoplasma gondii* (Romano et al., 2013) *in vitro*, and infection with *Chlamydia*-phage (Hsia et al., 2000). These stressors are often encountered by pathogens during infection *in vivo* (Wyrick, 2010). IFNγ, one of the stressors commonly studied, is a key component of immunity to intracellular pathogens. IFNγ induces expression of indoleamine-2, 3dioxygenase (IDO) that catalyzes the initial step in the degradation of L-tryptophan to *N*-formylkynurenine and kynurenine in eukaryotic cells (Beatty et al., 1994a). Such tryptophan depletion profoundly interferes with chlamydial growth, which, depending on the IFNγ concentration and exposure time, induces the bacteria to enter into a persistent growth form or results in bacterial eradication. Exposure to β-lactam antibiotics is another well-studied stressor that causes chlamydiae to enter into a persistent growth state and is used in simulating an inadequate antimicrobial treatment of chlamydiae infection (Matsumoto and Manire, 1970; Gerard et al., 2001; Giles and Wyrick, 2008; Carrasco et al., 2011; Wang et al., 2011a; Ouellette et al., 2012; Phillips-Campbell et al., 2014). *Chlamydia* spp. have a wide range of responses to stress. A clear shift of gene expression profiles specific to each stressor has been consistently found in many host cell types (Beatty et al., 1993, 1994b; Gerard et al., 2001, 2013; Molestina et al., 2002; Belland et al., 2003a,b; Ouellette et al., 2006; Lo et al., 2012). Changes in gene expression induced by stress at the levels of transcription and translation are consistent with the nature of a persistent growth form that undergoes DNA replication, but not division by binary fission. Interestingly, IFNγ exposure resulted in a global transcriptional upregulation lacking increased translation in chlamydiae (Ouellette et al., 2006). In contrast, penicillin exposure induced an alteration in transcription coupled to a change in translation (Ouellette et al., 2006). These data support that the impact of each stressor on bacteria is mediated by different mechanisms, and the bacterial response to each stress may be distinct.

**Figure 2:**
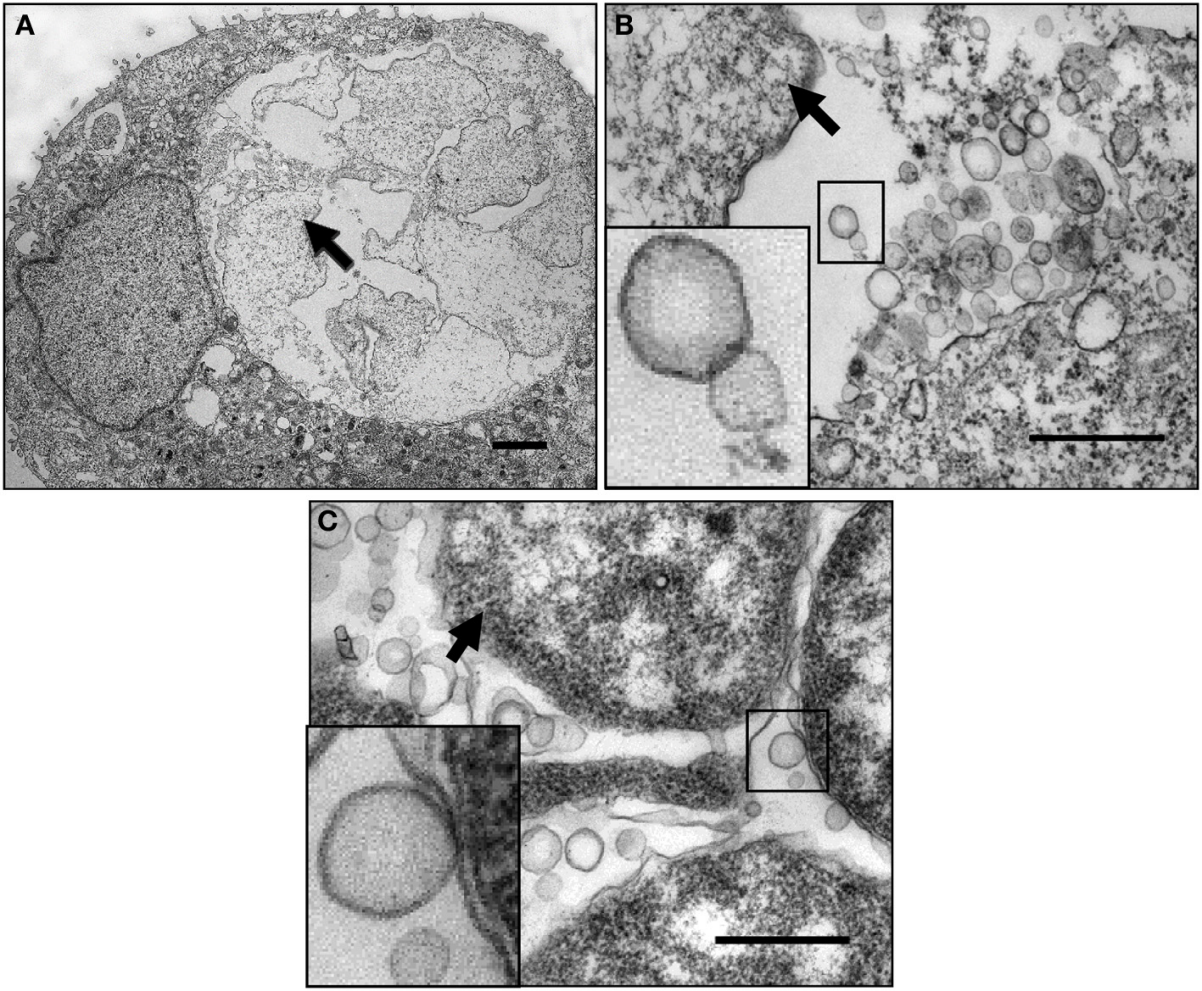
**Micrograph of chlamydial MVs in cultured human cervical epithelial cells. (A)** Ampicillin-induced persistent growth forms in infected human primary endocervical epithelial cells. **(B)** A high magnification image showing ampicillin-induced *C. trachomatis* MVs. The boxed MVs are enlarged in the insert. **(C)** IFNγ-induced *C. trachomatis* MVs in infected HeLa cells. The boxed bacterial membranes and the MVs are enlarged in the insert. Note: the vesicles appear to be single-membrane structures different from the double membrane structures of intact chlamydial organisms. They are associated with the bacterial surface, clustered or scattered within the inclusion lumen. Arrows indicate persistent forms. The primary cell cultures were established from endocervical tissue explants as previously described (Herbst-Kralovetz et al., 2008). Cells were infected with serovar F EBs resulting in a 30% infection rate. Ampicillin (10 μg/ml) was added to the culture at 16 h post-infection. For the IFNγ exposure model, HeLa cells were exposed to RPMI 1640 medium containing 50 units/ml of IFNγ for 24 h prior to infection. Fresh IFNγ-containing medium was added after infection. Cells were harvested at 36 h post-infection, fixed and processed for TEM as described previously (Belland et al., 2003a). Scale bars: **(A)** 500 nm. **(B,C)** 200 nm.

To explore the mechanisms by which *C. trachomatis* survives under persistence inducing conditions, we utilized human primary endocervical epithelial cells, the main site of *C. trachomatis* infection *in vivo*. We found that normal *C. trachomatis* forms could develop in cultured primary cells similarly to HeLa cells (Wang et al., 2011a). Figures 2A–C show that exposure to ampicillin or IFNγ led to aberrant RB phenotypes and an accumulation of abundant MVs generated by *C. trachomatis* in cultured cells. Additionally, confocal microscopy and cell fractionation analyses demonstrated that the secreted chlamydial T3S effectors, CopN and Tarp, and the secreted protease, CPAF (Zhong et al., 2001) were decreased in ampicillin-induced persistent forms (Wang et al., 2011a), the latter of which is consistent with previous observations using IFNγ exposure or iron-deprivation persistence models (Shaw et al., 2002; Heuer et al., 2003). Changes in the spectrum of host cytosolic chlamydial proteins may underlie the host-pathogen relationship because of their importance in modulating host cell signaling (Valdivia, 2008; Zhong, 2011; Mueller et al., 2014).

## MV produced during bacterial infection are present in *C. trachomatis* infected cells *in vivo* in humans

**Figure 3:**
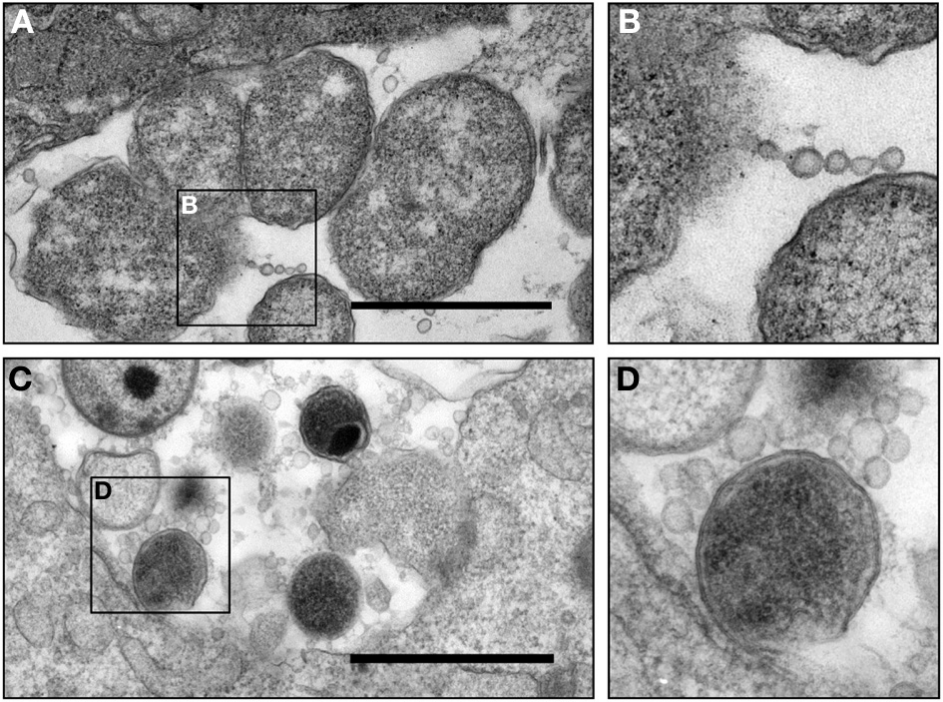
**Micrograph of chlamydial MVs in cervical epithelial cells from *C. trachomatis* infected patients. (A,B)** Presence of *C. trachomatis* vesicles associated with an aberrant *C. trachomatis* form in an inclusion. Vesicles appeared in the form of a chain or single round vesicular structures. This sample was obtained from a patient (patient 2) presented in Lewis et al. in this journal (Lewis et al., 2014); **(C,D)** presence of *C. trachomatis* MVs associated with a normal *C. trachomatis* form in an inclusion obtained from an epithelial cell from a second *C. trachomatis* infected patient. *C. trachomatis* infected human epithelial cells were collected by cytobrush from patients with an NAAT and culture positive *C. trachomatis* infection, and were immediately placed in a modified TEM fixative (Lewis et al., 2014) (currently under review for this Journal) and processed as previously described (Belland et al., 2003a). Scale bar = 500 nm.

We have been particularly intrigued by the observed vesicular structures that are derived from both pathogen and host cells during *C. trachomatis* infection in culture, as they may serve as a vital element for host-pathogen interactions. *C. trachomatis* is likely to encounter numerous known and not-yet-identified conditions *in vivo*. Of these, varying levels of IFNγ in the endocervix during chlamydial infection *in vivo* are likely crucial as demonstrated by studies *in vitro*, in animal models, and in observational studies in humans (Arno et al., 1990; Beatty et al., 1994a; Byrne, 2001; Aiyar et al., 2014; Lewis et al., 2014). The direct involvement of persistent growth forms in pathogenesis *in vivo* in humans is challenging to prove, but several TEM studies have visualized atypical pleomorphic RBs and aberrant *C. trachomatis* forms in individuals with chronic infections, in Fallopian tube tissues, and in the synovium of reactive arthritis patients (Patton et al., 1994; Nanagara et al., 1995; Mazzoli et al., 2000; Bragina et al., 2001). Very recently, the Quayle laboratory developed methodology to sample endocervical cells and components of the endocervical environment in *C. trachomatis* infected women. By using a cytobrush to retrieve cells followed by immediate placement of these brushes in modified TEM fixative, the ultrastructure of “*in vivo*” chlamydial growth forms in endocervical epithelial cells could later be visualized by TEM (Lewis et al., 2014). These TEM analyses revealed that chlamydial infection in the human endocervix could result in a variety of inclusion types, containing normal forms, a mixture of relatively “normal” and aberrant forms, or inclusions with highly aberrant forms, and this varies between patients (Lewis et al., 2014). Since MVs are so small in size and lack unique biological markers for identification by standard immunostaining techniques, we have begun to take advantage of these samples to examine MV production *in vivo*. High magnification TEM images were obtained from cross sections of several inclusions and indicated the *in vivo* presence of *C. trachomatis* MVs in inclusions sampled from two different patients (Figures 3A–D). MVs appeared to be single membrane structures and apparently associated with the OM of chlamydial organisms similar to those observed *in vitro* (Figure 2). The double membrane structures of *C. trachomatis* forms are intact, which is an indication of faithful sample preservation. This preliminary but novel observation provides an important link to those observations made *in vitro*, indicating that MVs are a component of *C. trachomatis* infection *in vivo* in humans. Further examination of samples using this methodology is in progress to determine the relationship between MV formation and the bacterial growth state in natural human infections.

## The importance of MVs during *C. trachomatis* infection

The best-studied mechanism that is related to OM changes in Gram-negative bacteria is the formation of MVs during both physiological adaptation and responses to envelope stress. Bacterial MVs are not only compositionally similar to the OM, containing LPS, phospholipids, and OM proteins, but also contain selective periplasmic or cytoplasmic components, such as toxins, DNAs, and RNAs, depending on the strain (Kadurugamuwa and Beveridge, 1999; Beveridge, 1999; Kuehn and Kesty, 2005). Research demonstrates the potential of MVs as vehicles of pathogenicity, as they can deliver complex bacterial molecules to target cells (Kuehn and Kesty, 2005; Bomberger et al., 2009; Amano et al., 2010; Elmi et al., 2012). The MVs also involve immune activation or suppression, stress responses, and attachment and internalization of the bacteria. Because of the cargo carrier nature and potent built-in adjuvanticity of most MVs studied to date, they are being utilized as vaccines (Collins, 2011; Unal et al., 2011). Engineered MVs in *E. coli* have exhibited a promising robust and tunable platform for the development of recombinant multivalent vaccines (Chen et al., 2010c; Bartolini et al., 2013).

Although chlamydial MVs were first observed over four decades ago (Matsumoto and Manire, 1970; Stirling and Richmond, 1980), the implications of these multi-functional MVs are only beginning to be elucidated (Giles et al., 2006; Giles and Wyrick, 2008; Wang et al., 2011a; Frohlich et al., 2012). A puzzle remains with regard to what role chlamydial MVs play during infection. Research data, including our own, suggest that the generation of chlamydial protein containing MVs occurs during productive infection and is enhanced by stress (Figures 2, 3) (Matsumoto and Manire, 1970; Giles et al., 2006; Giles and Wyrick, 2008; Wang et al., 2011a; Frohlich et al., 2012). The presence of MVs, not only during the β-lactam induced aberrant state, but also during both tryptophan starvation via IFNγ exposure and *in vivo* infection demonstrate a universal mechanism that appears to be responsive to stress rather than merely a by-product of dysfunctional membrane biogenesis during β-lactam exposure.

### Protein composition of chlamydial MVs

A challenge to studying chlamydial MVs is the obligate intracellular lifestyle of *C. trachomatis* and the complexity of isolating vesicle populations derived from the pathogen as opposed to the host cells. To more comprehensively understand the properties of chlamydial MVs, we developed a method that permits efficient isolation and enrichment of intact chlamydial MVs for biochemical analysis (Frohlich et al., 2012). This approach uses a combination of nitrogen cavitation, magnetic bead immunological capture, and isopycnic centrifugation of *C. trachomatis* infected L929 cells. This method not only allows for the study of MVs, but also isolation of chlamydial forms, host organelles, and host cytoplasm. Therefore, a “snapshot” of the total host and chlamydial environments can be obtained from a single experiment.

**Figure 4:**
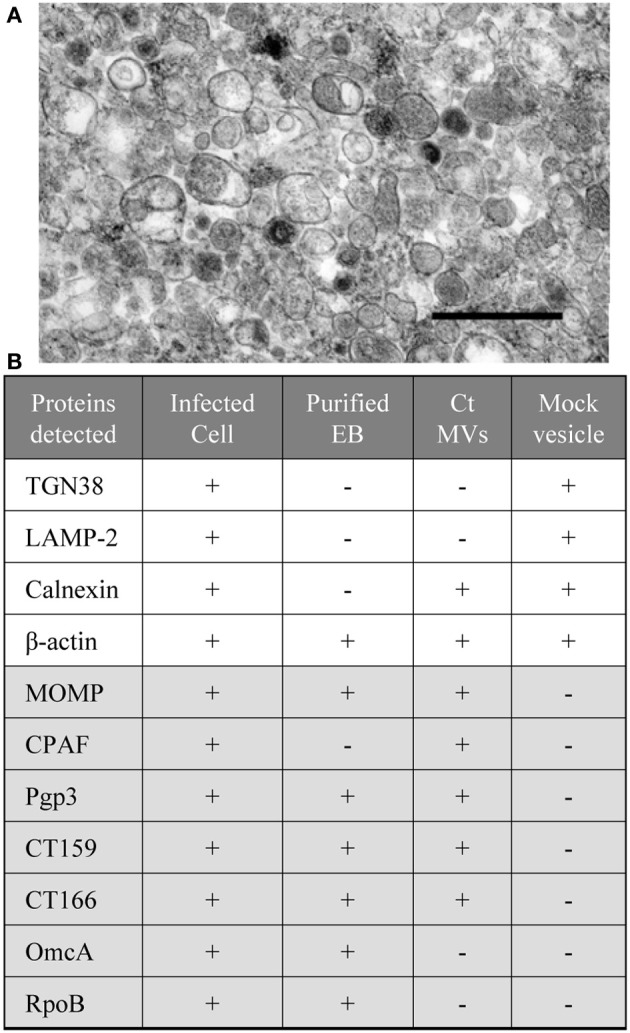
**Compositional analysis of *C. trachomatis* MV fractions. (A)** Micrographs of isolated MV fractions from *C. trachomatis* infected L929 cells by method described previously (Frohlich et al., 2012). At the ultrastructural level, the MVs show heterogeneity in their size and morphology. Scale bar = 500 nm; **(B)** *C. trachomatis* (Ct) or host cell proteins detected by immunoblotting with the lysates of infected cells, EBs, chlamydial MVs, and the control vesicles from mock infected cells. Note: coexistence of host ER marker (calnexin) with selective chamydial proteins (MOMP, CPAF, CT159, and CT160). Proteins were separated by 12% SDS-PAGE, followed by immunoblotting with antibodies to the protein of interest as described previously (Frohlich et al., 2012).

The integrity of chlamydial MVs isolated by this approach was confirmed by TEM analysis (Figure 4) (Frohlich et al., 2012). MOMP and several important *C. trachomatis* cytotoxic and/or secreted proteins (CPAF, Pgp3, CT159, and CT166) are found to be associated with MVs induced by ampicillin as determined by immunoblotting analyses (Figure 4) (Frohlich et al., 2012). These data support that MVs are generated by *C. trachomatis* as a means of carrying and delivering chlamydial proteins or antigens. Since those factors identified as being associated with chlamydial MVs often also stimulate traditional secretion pathways, it is possible that MV delivery provides an alternative and specific mode of protein delivery. It has been proposed that translocation of chlamydial proteases, CPAF and HtrA, from the bacterial cytoplasm to the periplasm is Sec-dependent (Wu et al., 2011; Zhong, 2011), but secretion of these proteins to an extrabacterial location or the host cytosol is likely mediated by MVs. One could envision a stress or developmental cycle-specific alternative protein delivery system employed to further circumvent host processes designed to limit chlamydial growth. Interestingly, evidence supports the association of chlamydial protein-containing MVs with the endoplasmic reticulum (Giles and Wyrick, 2008; Frohlich et al., 2012). Considering the widespread strategy of chlamydial exploitation of host cellular machinery, it is likely that the delivery of chlamydial proteins to the host by MVs, at least in some cases, hijacks the host cells' own protein delivery systems to provide a targeted and specific localization of chlamydial proteins. Further experimentation is needed to confirm and understand these processes.

### Potential functions of chlamydial MVs in infected host cells—cargo delivery

Although the exact destination of MVs is difficult to determine, TEM in combination with biochemical and immunodetection analyses suggest that MVs can connect with bacterial cells, accumulate in the inclusion lumen, associate with the inclusion membrane, evert from but still associate with the inclusion, and be found beyond the confines of the inclusion (Giles et al., 2006; Giles and Wyrick, 2008). Previous studies have suggested that a subset of these vesicles are antigen-containing structures that emerge from the inclusion membrane to release antigens from the inclusion without the need for inclusion disruption (Richmond and Stirling, 1981; Giles et al., 2006). These vesicles appeared to contain inclusion membrane proteins (Incs) co-localized with *C. trachomatis* antigens, including MOMP, LPS, GroEL2, and GroEL3, but not GroEL1 (Giles et al., 2006). We found that the isolated MV fraction was rich in CPAF, Pgp3, CT159, and CT166 (Frohlich et al., 2012). The functions of these proteins relating to chlamydial virulence or modulating host cellular functions have been studied (Belland et al., 2001; Zhong et al., 2001; Chen et al., 2010b; Thalmann et al., 2010). It is possible that distinct subpopulations are trafficked through cellular machinery alongside host vesicles. Presumably, the release of bioactive contents mediated by MVs influences the fate of host cells. How does MV translocation across the inclusion membrane and cytosol and/or surface of the host cell occur? How do these chlamydial antigen-containing vesicles influence host cell signaling and antigen presentation? Is the generation of MVs eliciting a variety of specific and highly regulated adaptive responses to protect *C. trachomatis* from the offending stress, or do they modulate innate and/or adaptive immunity? Do MVs produced by *C. trachomatis* relate to exosome formation by cervical epithelial cells with *C. trachomatis* infection? Certainly, any one of these events alone or in concert would confer a selective advantage in adaptation to and survival in the ever-changing intracellular niche.

### The potential role of MVs in the innate immune response

**Figure 5:**
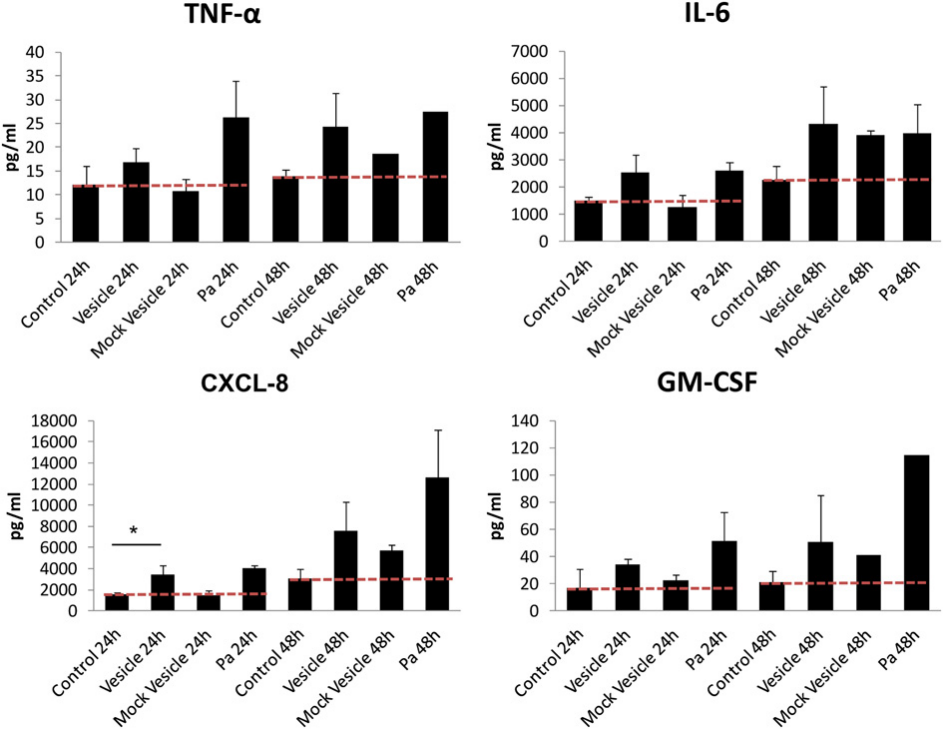
**Cytokine response of A2EN cells to vesicles isolated from a *C. trachomatis* infection of L929 cells**. MVs isolated from *C. trachomatis* infected cell cultures exposed to ampicillin (10 μg/ml) were added to a monolayer of A2EN cells. Purified host cell vesicles from a mock infection, heat-killed *Pseudomonas aeroginosa* PAO1 (Pa), and A2EN cells productively infected with chlamydial organisms were used as controls. Cytokine measurements were made using multiplex analyses as previously described (Buckner et al., 2013). ^*^p = 0.0230. The red dashed line represents baseline control values.

The MVs derived from many Gram-negative bacteria are heavily laden with complexes of pathogen-associated molecular patterns, such as LPS, flagellin, CpG DNA, and virulence factors. These MVs are strongly recognized by the host immune system, resulting in the upregulation of pro-inflammatory cytokine secretion (Amano et al., 2010; Kulp and Kuehn, 2010). To determine if chlamydial MVs, induced by ampicillin, elicit an innate immune response, we investigated the impact of isolated MVs on the secretion of cytokines by epithelial cells. An immortalized human endocervical epithelial cell line (A2EN) infected with *C. trachomatis* was used as our model of infection. A2EN cells retain site appropriate expression of hormone receptors, responsiveness to exogenous hormone stimulation, expression of TLRs, and responsiveness to TLR agonists by secreting cytokines, chemokines, and anti-microbial peptides (Buckner et al., 2013).MVs isolated from *C. trachomatis* infected cell cultures exposed to ampicillin were added to monolayers of A2EN cells. Purified host cell vesicles without *C. trachomatis* infection, heat-killed *Pseudomonas aeroginosa* PAO1, and A2EN cells productively infected with chlamydial organisms were used as controls. Culture supernatants were collected at 24 and 48 h. Chlamydial MVs induced a modest, albeit statistically significant (*p* = 0.0230 based on performing a One Way ANOVA with a Bonferroni post-test), 2-fold increase in CXCL-8 at 24 h compared to the mock MV control, although, no significant increase of IL-6, TNF-α, or GM-CSF was observed (Figure 5). At 48 h, however, the differences observed were not significant as both chlamydial MVs and the mock vesicle control induced a similar increase in CXCL-8 and GM-CSF levels, perhaps due to the species differences between the mouse L929 derived vesicles and the human A2EN cells. As previously published, *C. trachomatis* infection in A2EN cells failed to upregulate secretion of CXCL8 (Buckner et al., 2013). Productive infection with *C. trachomatis* conducted at the same time as the MV experiments recapitulated these published observations and provided an additional control lending to the consistent response of A2EN cells across different laboratories (data not shown). These data suggest that MVs have more capacity to elicit an innate immune response than productive chlamydial infection, and that the molecules on the surface or contained within these vesicles may be structurally distinct compared to those present on or within whole chlamydial particles. The significance of such an early (at 24 h) but weak stimulatory effect on endocervical cells by chlamydial MVs remains to be determined. These results are clearly different from previously reported observations with MVs from other Gram-negative bacteria, as MVs derived from these other bacteria induce a robust and significant increase in pro-inflammatory cytokines and chemokines and induce antibody production (Amano et al., 2010; Nakao et al., 2011; Sharpe et al., 2011; Zhao et al., 2013). However, given the obligate intracellular nature of *C. trachomatis*, a robust immune response would not likely be favorable to continued or long-term infection. These results support other observations of immune-evasion tactics used by chlamydiae, even in the delivery of MVs.

### The potential role of MVs in exchange of genetic materials

It has been demonstrated that bacterial MV production provides a general advantage for survival and social networks in many bacteria species (Kuehn and Kesty, 2005; Amano et al., 2010; Bielig et al., 2011; Unal et al., 2011). Of great interest to the study of MVs is their participation in inter- and intra-species exchange of bacterial material in addition to their immuomodulatory functions (Kadurugamuwa and Beveridge, 1999; Renelli et al., 2004; Chiura et al., 2011; Barteneva et al., 2013). MVs may bind to bacterial or host cells, fuse with the target cell membrane, and deliver their cargo to the cytosol of the target cells. Recent evidence that protein and DNA transfer between distantly related species raises the prospect of a widely distributed mechanism of bacterial communication (Dubey and Ben-Yehuda, 2011). This poses an interesting potential for *C. trachomatis* given the vast sampling of both normal and pathogenic microorganisms potentially inhabiting the same niche in the genital tract and could potentially provide a significant adaptation and survival advantage.

While there is no evidence that chlamydial MVs deliver products to other species, it is possible that MVs could be used to deliver not only proteins but also genetic materials among chlamydiae. Recently, it has become evident that *Chlamydia* spp. have all the necessary recombination machinery despite an inclusion-confined lifestyle (Zhang et al., 1995; Demars et al., 2007; Joseph et al., 2011; Harris et al., 2012). Recombination has been documented between strains with different tissues tropisms, the lymphogranuloma venereum with the urogenital biovars, and within different genital strains *in vitro* and *in vivo* (Jeffrey et al., 2010, 2013; Harris et al., 2012). Whole genome sequencing analyses with a collection of diverse clinical isolates and laboratory strains have revealed that the recombination or mutation events often occur in several regions of the chromosome encoding surface-exposed proteins, such as MOMP, Pmps and the T3S system effector Tarp (Gomes et al., 2004; Brunelle and Sensabaugh, 2006; Gomes et al., 2006; Joseph et al., 2011; Harris et al., 2012). Different *C. trachomatis* strains can recombine following mixed infection for a relatively short time period *in vitro* (Jeffrey et al., 2013). Their potential capabilities for DNA exchange might explain, at least in part, the acquisition of virulence genes and fitness traits, as well as variations in these surface exposed proteins. Whether or not MVs directly contribute to the horizontal gene transfer between chlamydial organisms, as is observed in other bacteria, is unknown, but it begs the question: what impact does MV-mediated cell-to-cell communication have on pathogenesis and bacterial survival within the host? How might these MVs directly or indirectly affect the genital tract microbiome? Further addressing these questions will help in understanding the occurrence of antigen variation and diversity in *C. trachomatis* during infection and promote the development of novel genetic tools to study genetics and pathogenesis of *C. trachomatis.*

## Molecular mechanisms controlling MV formation in *C. trachomatis*

Despite tremendous efforts, mechanisms by which bacteria produce MVs while sustaining their viability remain unclear. Based on studies in model organisms, including *P. aeruginosa* and *E. coli*, several mechanisms of MV formation have been proposed. First, the loss of OM-PG cross-links may allow excess lipid formation to induce MV formation. Second, accumulated periplasmic proteins simply push out the OM, trapping large amounts of protein inside the resulting vesicle. Third, the involvement of integral membrane proteins or signal molecules [such as *Pseudomonas* quorum sensing (PQS) molecules] induces membrane curvature and vesiculation (Schertzer and Whiteley, 2012). Fourth, the presence of rough LPS in bacteria contributes directly and indirectly to the formation of MVs (Kadurugamuwa and Beveridge, 1995; Sabra et al., 2003). Finally, genetic mechanisms may exist to regulate MV formation (McBroom et al., 2006; Schwechheimer et al., 2013). These MV inducing factors that have been proposed may not be mutually exclusive, and multiple dynamic surface components may contribute to MV production in bacteria (Kulp and Kuehn, 2010; Schwechheimer et al., 2013).

We know surprisingly little about OM biogenesis, and even less is known about the related vesicle formation in *C. trachomatis*. It is likely that after synthesis in the bacterial cytoplasm, chlamydial protein translocation to the OM involves complex steps conserved in Gram-negative bacteria (Hagan et al., 2010; Silhavy et al., 2010). These include: (i) translocation across the IM via the Sec pathway; (ii) folding facilitated by periplasmic chaperones/proteases, and (iii) assembly and insertion into the lipid phase of the OM, a process facilitated by a β-barrel assembly machine (BAM) complex that consists of Omp85 and its interacting lipoproteins (Ricci and Silhavy, 2012). Also necessary for maintenance of EB envelope integrity is the disulfide bond cross-linking of MOMP with OmcA and OmcB (Hatch, 1996).

We hypothesize that both bacterial and host factors contribute to the generation and release of MVs from *C. trachomatis*. We envision different mechanistic scenarios involved in MV formation during productive infection or the persistent growth state induced by ampicillin. In the case of productive infection, MV generation may reflect local OM deformation and/or a physiological turnover of envelope components because of envelope remodeling, a critical process in chlamydial development. In fact, RB multiplication and division require a rapid surface expansion or alteration achieved by the synthesis, assembly, and insertion of lipid or non-lipid OM components, while transition from a large RB to a small EB undergoes the opposite. In contrast, ampicillin induced hyper-vesiculation phenotypes may be the result of an envelope stress response. Exposure to ampicillin blocks RB division, although DNA replication remains. These can, in turn, result in attenuation of OM protein expression, accumulation of misfolded proteins in the periplasm, activation of proteolytic processes, and interference with the correct assembly and insertion of proteins in the OM. All of these are likely to induce heightened vesiculation. MV formation may offer a means to remove stress caused by the accumulated “toxic” waste or unfolded proteins. Chlamydial HtrA, a key player in envelope stress response (Huston et al., 2007, 2008; Zhong, 2011), is likely to play a role in controlling the formation of MVs, as is observed in other bacteria (McBroom et al., 2006). Given the strong capacity of chlamydial exploitation of host cellular machinery, MV cargo delivery may partially depend on a yet to be defined host trafficking pathway. Whether or not a potential host factor contributes to the formation of chlamydial MVs remains to be determined. Nevertheless, studying molecular mechanisms underlying envelope adaptation to development or stress offers a powerful tool and new route to understanding how *C. trachomatis* adapts to and survives in nutrient rich but hostile intracellular niches.

## Summary and perspective

The production of MVs in bacteria is a universal mechanism whereby virulence factors, signaling molecules, and genetic materials can be packaged and effectively transported to target cells. Major challenges in the field of vesicle research with obligate intracellular bacteria include (i) developing new comprehensive approaches for vesicle isolation and characterization, (ii) isolating pure populations from bacteria or host cells, and (iii) monitoring vesicle dynamics under physiologic relevant conditions. Nevertheless, the information already available indicates that MVs produced during chlamydial infection are present in cultured cell lines, primary human endocervical epithelial cells infected with *C. trachomatis*, and more importantly, in clinical specimens from *C. trachomatis* infected patients. These data provide preliminary evidence that the MV is a component of chlamydial infection *in vivo*. We hypothesize that *C. trachomatis* varies OM organization and produces MVs during the developmental cycle and in response to stress. Many mysteries remain with regard to how MVs contribute to host-pathogen interactions, the relationship of developmental envelope biogenesis within the context of MV formation and regulation, and the modes of MV cellular targeting and delivery. Improved understanding of the mechanisms of MV mediated mass material exchanges and the effects on *C. trachomatis* survival may have relevance to several important aspects of chlamydial biology, including the *C. trachomatis* persistent growth state *in vivo*. Recent progresses in chlamydial genetics may have great potential to inspire significant advance in MV studies in *C. trachomatis* (Wang et al., 2011b; Gérard et al., 2013; Gong et al., 2013; Song et al., 2013; Bauler and Hackstadt, 2014). Since global control of *C. trachomatis* infection will best be achieved with a vaccine, developing a greater understanding of the functional role and the mechanisms of envelope modification and MV production may open new therapeutic avenues against this medically important intracellular bacterium.

### Conflict of interest statement

The authors declare that the research was conducted in the absence of any commercial or financial relationships that could be construed as a potential conflict of interest.
